# Sodium Butyrate Reduces Colitogenic Immunoglobulin A-Coated Bacteria and Modifies the Composition of Microbiota in IL-10 Deficient Mice

**DOI:** 10.3390/nu8120728

**Published:** 2016-11-24

**Authors:** Tenghui Zhang, Chao Ding, Mingli Zhao, Xujie Dai, Jianbo Yang, Yi Li, Lili Gu, Yao Wei, Jianfeng Gong, Weiming Zhu, Ning Li, Jieshou Li

**Affiliations:** 1 Department of General Surgery, Jinling Hospital Affiliated to Southern Medical University, No. 305 East Zhongshan Rd., Nanjing 210002, China; tenghuiff@163.com (T.Z.); zhao_ming_li@163.com (M.Z.); 2 Department of General Surgery, Jinling Hospital, Medical School of Nanjing University, Nanjing 210002, China; dingchao19910521@126.com (C.D.); d670677094@163.com (X.D.); yang_jianbo@126.com (J.Y.); liyi1860@126.com (Y.L.); drgulili@163.com (L.G.); dr_betty@126.com (Y.W.); dr_zhuweiming@126.com (W.Z.); proflining@163.com (N.L.); lijieshou@163.com (J.L.)

**Keywords:** sodium butyrate, immunoglobulin A-coated bacteria, microbiota composition, colitis, IL-10^−/−^ mice

## Abstract

High levels of immunoglobulin A (IgA)-coated bacteria may have a role in driving inflammatory bowel disease (IBD). We therefore investigated the effect of sodium butyrate on microbiota in IBD prone interleukin (IL)-10^−/−^ mice. At 8 weeks of age, mice were allocated into three groups (*n* = 4/group): normal (C57BL/6), IL-10^−/−^, and IL-10^−/−^ treated with sodium butyrate (100 mM). Severity of colitis, inflammatory cytokine and short-chain fatty acid (SCFA) concentration in proximal colon contents, the percentage of IgA-coated bacteria and microbiota composition by 16S ribosomal RNA assessment of stool were measured after 4 weeks of treatment. Sodium butyrate ameliorated histological colitis and decreased levels of tumor necrosis factor (TNF)-α and IL-6 in IL-10^−/−^ mice compared with those without treatment. At the phylum level, a reduction in *Bacteroidetes* and an increase in *Firmicutes* in IL-10^−/−^ mice treated with sodium butyrate were observed. Additionally, *Prevotellaceae* species were reduced in IL-10^−/−^ mice treated with sodium butyrate as compared with those without treatment. The level of biodiversity was slightly increased and the amount of IgA-coated bacteria decreased in IL-10^−/−^ mice treated with sodium butyrate compared with those without treatment. Our results indicate that sodium butyrate protects against colitis, possibly through modifying the gut microbiota, enriching biodiversity and reducing the amount of colitogenic IgA-coated bacteria in IL-10^−/−^ mice.

## 1. Introduction

Altered composition of the gastrointestinal tract microbiota has been widely recognized as playing a major role in the pathogenesis of inflammatory bowel disease (IBD) [1,2,3], although the etiology of IBD remains largely unknown. Many studies consistently report a change in biodiversity, such as a reduction of the *Firmicutes* phylum in Crohn’s disease (CD) patients [4,5] and an increase of *Enterobacteriaceae* in IBD patients and mouse models [6]. Moreover, studies have observed individuals with IBD had increased bacterial translocation [7,8] which has been proposed to trigger immune activation and inflammation. Furthermore, it has been found that the colitogenic bacteria contributing to disease severity or exacerbation of inflammation in IBD might be identified by high levels of immunoglobulin A (IgA) coating [9]. Secretory IgA, the predominant antibody isotype produced at mucosal surfaces, can not only provide protection against infection by binding pathogens, but also protect against mucosal penetration by commensals [10,11]. However, drugs or molecules modulating IgA-coated bacteria in the intestinal microbiota have never been investigated.

Among the most important environmental factors impacting microbial composition is diet, which has been demonstrated to influence microbiome composition throughout mammalian evolution [12]. It has been reported that Western-style diets, which are low in fiber, decrease beneficial *Firmicutes* and increase mucosa-associated *Proteobacteria* compared with a high fiber diet [13]. Additionally, the anti-inflammatory efficacy of dietary fibers in a murine colitis model of IBD has been indicated in many studies [14,15,16]. The effect of dietary fibers or synbiotics on the prevention of bacterial translocation in either experimental mice or humans also has been reported. Short-chain fatty acids (SCFAs), particularly acetate, propionate, and butyrate, are the products of fermented soluble dietary fiber. Sodium butyrate plays a crucial role as a fuel source for intestinal epithelial cells [17] and exerts effects on both gut morphology and function [18]. SCFAs contribute to normal large bowel function and prevent pathology through their modification by commensal microbiota and their metabolism by colonocytes. Depletion of sodium butyrate-producing bacteria in IBD microbiota is clearly evidenced by a reduction in sodium butyrate-producing metabolic pathways [19,20], as well as in concentrations of fecal sodium butyrate [21,22]. Moreover, one study found that sodium butyrate regulates the size and function of the colonic Treg pool and protects against colitis in a GPR43-dependent manner in mice [23]. These results suggest that sodium butyrate reduces colitis by modulating the function and composition of both gut microbiota and leukocytes in IBD.

Thus, we hypothesized that sodium butyrate could ameliorate colitis by modulating the colonic commensal microflora, especially levels of IgA-coated bacteria. The objective of this study was to evaluate whether sodium butyrate would reduce colitis severity through modulation of microbiota in the interleukin (IL)-10^−/−^ mouse model of experimental IBD.

## 2. Materials and Methods

### 2.1. Animals

Both IL-10^−/−^ and wild-type mice (8 weeks old) on a C57BL/6 background were obtained from the Jackson Laboratory (Bar Harbor, ME, USA). Mice were bred and maintained in specific pathogen-free conditions at the Model Animal Research Centre of Nanjing University (Nanjing, China). The experimental procedures were performed in accordance with the Guidelines for Animal Experiments at Jinling Hospital and were approved by the Ethics Committee at Jinling Hospital (2014NLY-112). The mice were maintained on a 12-h dark-light cycle and allowed free access to food and tap water under controlled temperatures.

### 2.2. Drug Administration Protocol

Mice were coded and randomized into three groups (4 per group): C57BL/6 mice (control, tap water), IL-10 group (IL-10^−/−^ mice, tap water), and the IL-10 + sodium butyrate group (sodium butyrate (Sigma-Aldrich, Shanghai, China) (100 mM: 1.1 g sodium butyrate dissolved in 100 mL water) administered orally in drinking water, with water bottles changed every 5 days). An equal number of males and females were used in each group. The mice were anesthetized by intraperitoneal administration of ketamine (0.3 mL/100 g body weight) and euthanized 4 weeks after treatment. All samples were immediately frozen and stored at −80 °C excepted for proximal colons for histology.

### 2.3. Histology and Colitis Scores

Proximal colons were excised and cleaned with Dulbecco’s phosphate buffered saline prior to fixation in 4% paraformaldehyde and then processed by routine paraffin embedding, sectioning and hematoxylin and eosin staining. Colitis scores were determined by two independent pathologists who were blinded to the experimental parameters. Each proximal colon segment was scored from 0 to 4 using well-established criteria [24]. The summation of scores per mouse provided a total colonic disease score.

### 2.4. Measurement of SCFAs

Samples of proximal colon content were collected immediately after the animals were euthanized and stored at −80 °C. The measurement of SCFAs was performed as described previously [25].

### 2.5. Quantitative Real-Time RT-PCR

Quantitative real-time PCR was performed with proximal colon as described previously [26] with primers specific for tumor necrosis factor (TNF)-α and IL-6. Relative expression was calculated using the 2^-ΔΔCt^ method after normalizing to the housekeeping gene glyceraldehyde phosphate dehydrogenase (GAPDH). The sequences of the primers used were: TNF-α: 5′-CCTCTCTCTAATCAGCCCTCTG-3′, 5′-GAGGACCTGGGAGTAGATGAG-3′; IL-6: 5′-ACTCACCTCTTCAGAACGAATTG-3′, 5′-CCATCTTTGGAAGGTTCAGGTTG-3′; GAPDH: 5′-AGGCCGGTGCTGAGTATGTC-3′, 5′-TGCCTGCTTCACCACCTTCT-3′.

### 2.6. Fecal IgA-Coated Bacteria Flow Cytometry

For detection of IgA-coated bacteria, we used a previously reported method [9]. Briefly, Fast Prep Lysing Matrix D tubes containing ceramic beads (MP Biomedicals, Santa Ana, CA, USA) were used for homogenization and PE-conjugated anti-mouse IgA (clone mA-6E1, 1:12.5 dilution; eBioscience, San Diego, CA, USA) was used for staining prior to flow cytometric analysis (Epics Altra, Beckman Coulter, Indianapolis, IN, USA).

### 2.7. High Throughput Sequencing of Microbiota Using 16S rRNA PCR

Bacterial DNA was extracted and 16S rRNA gene amplicons were generated and sequenced on an Illumina MiSeq (Illumina Inc., San Diego, CA, USA) with MiSeq Control Software v. 2.2.0 (Illumina Inc., San Diego, CA, USA). Sequence data were processed and analyzed using QIIME. PCR amplification of the V5-V4 regions of the bacterial *16S rRNA* gene was performed using universal primers (515F 5′-GTGCCAGCMGCCGCGGTAA-3′and 907R 5′-CCGTCAATTCMTTTRAGT-3′) incorporating a unique sample barcode sequences. For amplicon library preparation, 20 ng of each genomic DNA, 1.25 U Taq DNA polymerase, 5 μL 10 × Ex Taq buffer (Mg^2+^ plus), 10 mM dNTPs (all reagents purchased from TaKaRa Biotechnology (Dalian) Co., Ltd, Dalian, China), and 40 pmol primer mix was used per 50 μL amplification reaction. The PCR condition is as follows: 5 min initial denaturation at 95 °C; 28 cycles of denaturation at 95 °C (30 s), annealing at 55 °C (30 s), elongation at 72 °C (45 s), and final extension at 72 °C for 7 min. The PCR products were purified with magnetic beads (Axygen^®^, NewYork, NY, USA). Amplicon library concentration was estimated with the 2100 Bioanalyzer System (Agilent Technologies^®^, Santa Clara, CA, USA), and equal amount of amplicon from each samples was pooled together. Then the emulsion PCR and sequencing were performed on Ion Torrent Personal Genome Machine (PGM) platform following the manufacturer’s recommendations.

Our strategy was a dual-index paired-end sequencing approach. The fusion primers were designed to include the appropriate P5 and P7 Illumina adapter sequences, an eight nucleotide index sequence, and the gene-specific primer. A dual-index sequencing approach allowed us to generate a large number of high-quality sequences while minimizing the cost of long and customized primers [27]. 

### 2.8. Statistical Analyses

Statistical analyses were performed using SPSS software version 20.0 (IBM, Armonk, NY, USA). Data are expressed as means with their standard deviation. One-way ANOVA was used for multiple comparisons, and results were considered statistically significant if *p*-values were < 0.05.

## 3. Results

### 3.1. Sodium Butyrate Ameliorated Histological Colitis, Which Was Associated with Decreased Proinflammatory Cytokines in IL-10^−/−^ Mice

**Figure 1:**
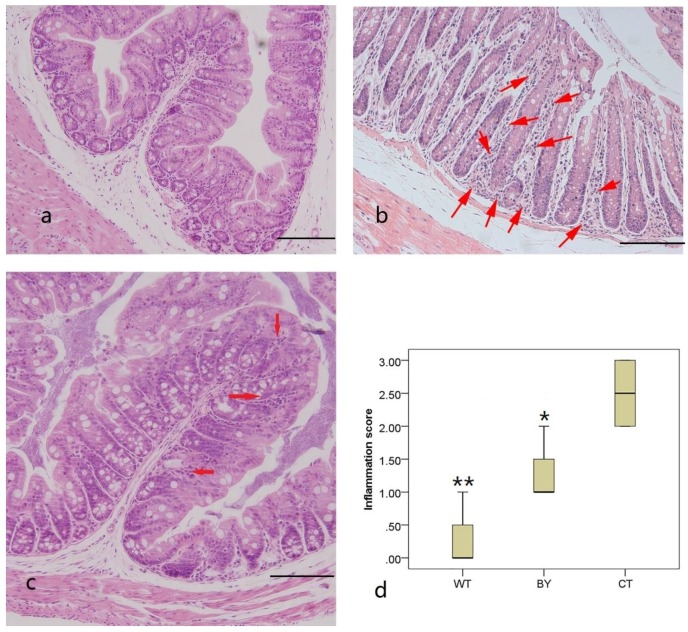
Histological features and scores of the proximal colons from mice in three groups. Representative HE stained sections from three groups (*×*200 magnification) are shown. Wild type mice (WT and (**a**)), IL-10^−/−^ mice without treatment (CT and (**b**)), interleukin (IL)- 10^−/−^ mice treated with butyrate (BY and (**c**)). Arrows indicate infiltration of inflammatory cells. Histological scores of all three groups (**d**). Data are presented as mean ± SEM (*n* = 4 for each group, Bars = 100 μm. * *p* < 0.05 and ** *p* < 0.01 versus IL-10^−/−^ mice without treatment).

First, we evaluated the protective effects of sodium butyrate treatment on colitis severity. Compared with wild-type mice, IL-10^−/−^ mice without treatment showed greater infiltration of inflammatory cells in colonic mucosa and higher mean histological scores (2.5 ± 0.58 vs. 0.25 ± 0.50, *p* = 0.001). However, IL-10^−/−^ mice receiving sodium butyrate treatment demonstrated a significant reduction in inflammatory cells in the colon, as shown by reduced inflammatory cell infiltration and lower mean inflammatory scores (1.25 ± 0.50 vs. 2.5 ± 0.58, *p* = 0.025). There were no significant differences between the IL-10^−/−^ mice receiving sodium butyrate and wild-type mice (Figure 1).

**Figure 2:**
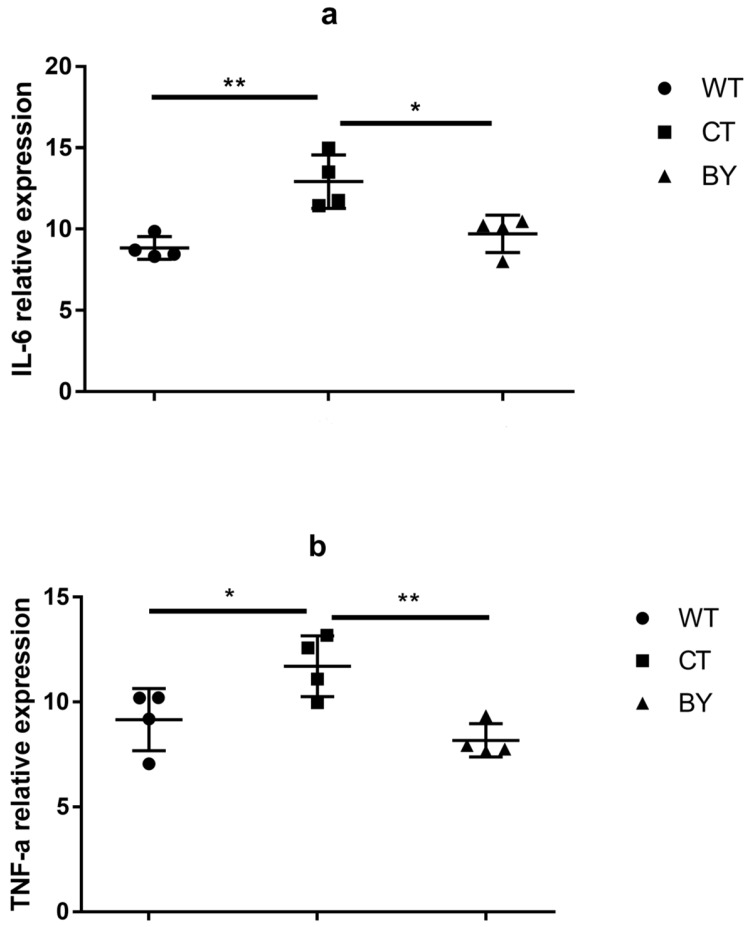
The relative expression of colitis inflammatory factors IL-6 (**a**) and tumor necrosis factor (TNF)-a (**b**) of the proximal colons in three groups of mice. Wild-type mice (WT), IL-10^−/−^ mice without treatment (CT), IL-10^−/−^ mice treated with butyrate (BY); Data are presented as mean ± SEM (*n* = 4 for each group, * *p* < 0.05 and ** *p* < 0.01 versus CT group).

Next, we used qRT-PCR to quantify TNF-α and IL-6 mRNA of proximal colon in different groups. As shown in Figure 2, the IL-10^−/−^ mice without treatment demonstrated higher expression of IL-6 and TNF-α than controls (12.92 ± 1.64 vs. 8.84 ± 0.70, *p* = 0.001; 11.71 ± 1.45 vs. 9.16 ± 1.48, *p* = 0.014, respectively). The IL-10^−/−^ mice receiving sodium butyrate treatment had a significantly lower level of IL-6 and TNF-α than the IL-10^−/−^ mice without treatment (9.71 ± 1.15 vs. 12.92 ± 1.64, *p* = 0.006; 8.17 ± 0.79 vs. 11.71 ± 1.45, *p* = 0.002, respectively), which was comparable to controls (*p* = 0.388 and *p* = 0.285, respectively). 

### 3.2. The impact of Sodium Butyrate on the Concentrations of SCFAs in Proximal Colon Contents

**Figure 3:**
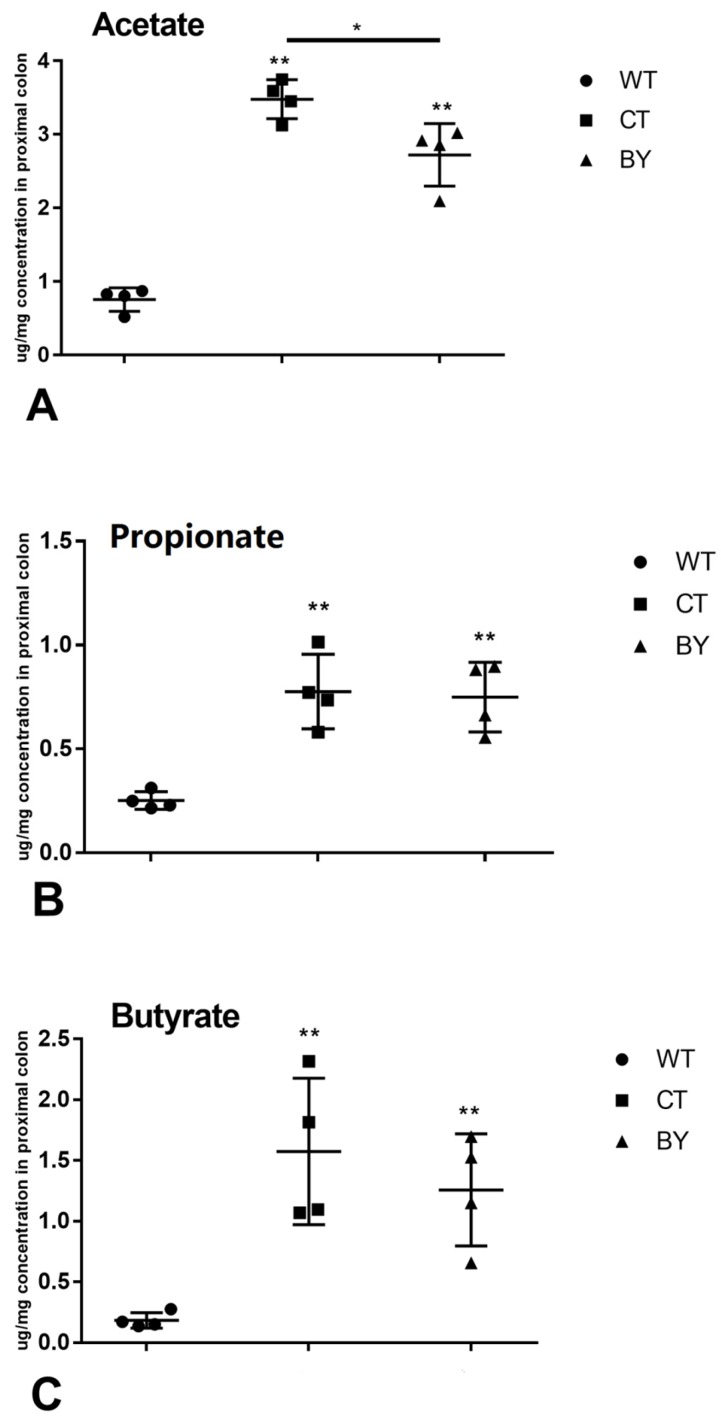
The acetate (**A**), propionate (**B**) and butyrate (**C**) of concentration in proximal colon contents in three groups of mice. Wild-type mice (WT), IL-10^−/−^ mice without treatment (CT), IL-10^−/−^ mice treated with butyrate (BY); Data are presented as mean ± SEM (*n* = 4 for each group, * *p* < 0.05 and ** *p* < 0.01 versus WT group).

Compared with wild-type mice, concentrations of acetate, propionate and sodium butyrate were significantly increased in IL-10^−/−^ mice treated with or without sodium butyrate compare to the wild-type mice (acetate: 3.48 ± 0.27 vs. 0.76 ± 0.16 μg/mg, *p* < 0.001 and 2.72 ± 0.42 vs. 0.76 ± 0.16 μg/mg, *p* < 0.001; propionate: 0.78 ± 0.18 vs. 0.25 ± 0.04 μg/mg, *p* = 0.001 and 0.75 ± 0.17 vs. 0.25 ± 0.04 μg/mg, *p* = 0.001; sodium butyrate: 1.57 ± 0.60 vs. 0.18 ± 0.06 μg/mg, *p* = 0.044 and 1.26 ± 0.46 vs. 0.18 ± 0.06 μg/mg, *p* = 0.042, respectively). There were no significant differences in SCFAs concentration between IL-10^−/−^ mice treated without and with sodium butyrate, except for the acetate concentration (3.48 ± 0.27 vs. 2.72 ± 0.42 μg/mg, *p* = 0.006) (Figure 3).

### 3.3. Effects of Sodium Butyrate on the Composition of Microbiota

**Figure 4:**
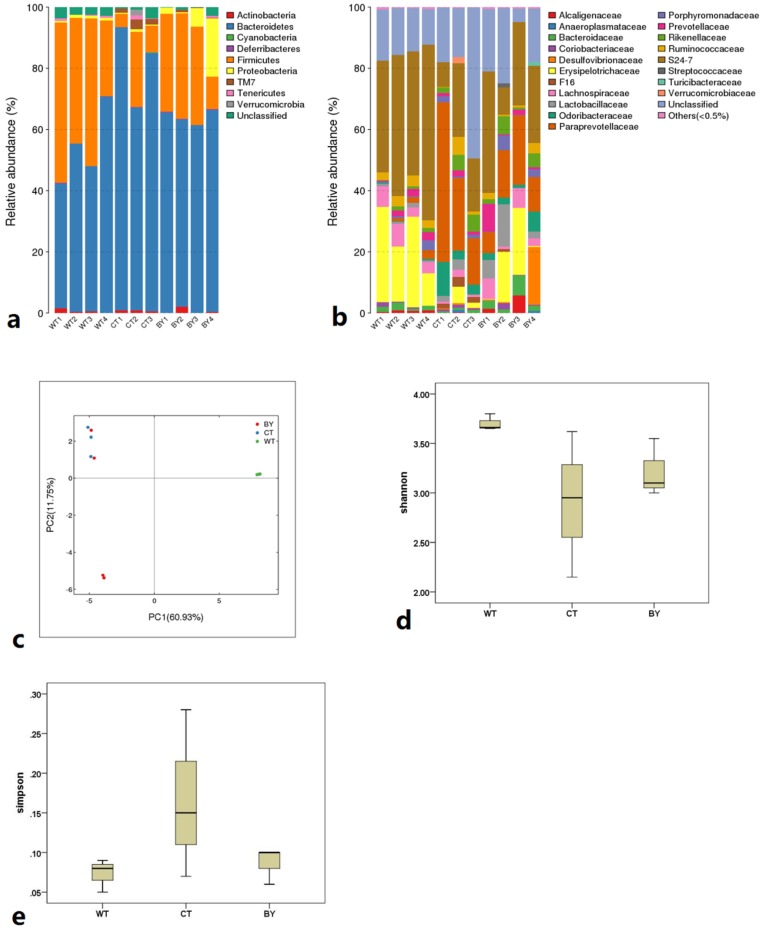
The microbiota composition of proximal colon contents in three groups of mice. The microbiota composition at the phylum level (**a**) and the family level (**b**) in three groups, the principal component analysis in three groups (**c**), the shannon index (**d**) and the simpson index (**e**) of biodiversity in three groups, Wild-type mice (WT), IL-10^−/−^ mice without treatment (CT), IL-10^−/−^ mice treated with butyrate (BY); Data are presented as mean ± SEM (* *p* < 0.05 and ** *p* < 0.01 versus CT group).

Sequencing of 16S ribosomal RNA gene tags from stool samples identified the dominant microbiota in all groups to be composed primarily of 2 bacterial phyla, *Firmicutes* and *Bacteroidetes*, with minor amounts of *Tenericutes* and *Proteobacteria* detected (Figure 4). At the phylum level, compared with IL-10^−/−^ mice without treatment, a reduction in *Bacteroidetes* (62.76 ± 2.38 vs. 81.07% ± 13.51%, *p* = 0.046) and an increase in *Firmicutes* (32.86 ± 1.47 vs. 12.68% ± 10.62%, *p* = 0.012) in IL-10^−/−^ mice treated with sodium butyrate was observed. Furthermore, the amount of *Firmicutes* in IL-10^−/−^ mice treated with sodium butyrate was significantly lower than wild-type mice (32.86 ± 1.47 vs. 47.22% ± 5.69%, *p* = 0.046) but the amount of *Bacteroidetes* was not different (62.76 ± 2.38 vs. 47.73 ± 7.19, *p* = 0.085).

Additionally, at the family level, *Lactobacillaceae* and *Erysipelotrichaceae* were increased in IL-10^−/−^ mice treated with sodium butyrate as compared with those without treatment (6.69 ± 6.75 vs. 1.81 ± 1.64%, *p* = 0.187 and 12.87 ± 11.27 vs. 2.38% ± 2.65%, *p* = 0.155), although there were no significant differences. At the genus level, the amount of *Prevotella* in IL-10^−/−^ mice without treatment was significantly higher than wild-type mice (31.68 ± 19.23 vs. 2.34% ± 1.87%, *p* = 0.021), but was reduced in IL-10^−/−^ mice treated with sodium butyrate (31.68 ± 19.23 vs. 18.79% ± 16.18%, *p* = 0.219). There were no significant differences between IL-10^−/−^ mice treated with sodium butyrate and wild-type mice (*p* = 0.131). We also found that sodium butyrate-producing *Eubacterium* bacteria in wild-type mice was significantly higher than other groups (25.67 ± 11.24 vs. 0.00, *p* = 0.003; 25.67 ± 11.24 vs. 0.00, *p* = 0.003). However, sodium butyrate-producing *Clostridium* bacteria in wild-type mice was lower than IL-10^−/−^ mice without treatment (0.00 vs. 46.00 ± 42.04, *p* = 0.060; 0.00 vs. 2.33 ± 2.31, *p* = 0.910). Furthermore, there were no significant differences of *Roseburia* in groups.

The principal component analysis found that the similarity in both wild-type mice and IL-10^−/−^ mice without treatment was high, although one sample in IL-10^−/−^ mice treated with sodium butyrate had a low similarity. The diversity analysis showed that the abundance of microbiota in IL-10^−/−^ mice treated with sodium butyrate was slightly higher than those without treatment (Shannon index: 3.22 ± 0.29 vs. 2.91 ± 0.74, *p* = 0.441; Simpson index: 0.09 ± 0.02 vs. 0.17 ± 0.11, *p* = 0.175), but the biodiversity in wild-type mice also tended to be higher than in IL-10^−/−^ mice treated with sodium butyrate (Shannon index: 3.70 ± 0.08 vs. 3.22 ± 0.29, *p* = 0.243; Simpson index: 0.07 ± 0.02 vs. 0.09 ± 0.02, *p* = 0.806). However, none of these differences were statistically significant.

### 3.4. Sodium Butyrate Reduced the Amount of IgA-Coated Bacteria

**Figure 5:**
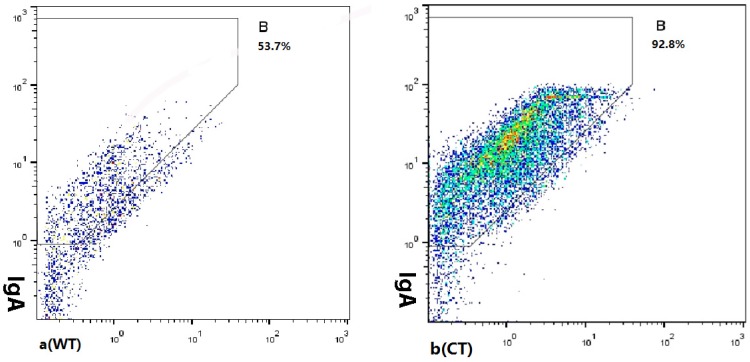

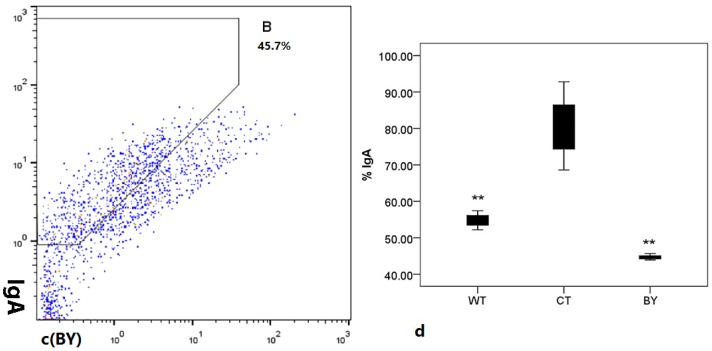
The IgA coating bacteria detected by the Flow Cytometry and the percent of IgA coating bacteria (**d**) in proximal colon contents in three groups of mice. Wild-type mice ((**a**), WT), IL-10^−/−^ mice without treatment ((**b**), CT), IL-10^−/−^ mice treated with butyrate ((**c**), BY); Data are presented as mean ± SEM (*n* = 4 for each group, * *p* < 0.05 and ** *p* < 0.01 versus CT group).

IgA-coated bacteria were previously found to have a role in driving IBD and targeted elimination of such bacteria may reduce, reverse, or even prevent disease development [9]. Therefore, we measured the levels of IgA-coated bacteria in mouse feces using flow cytometry (Figure 5). The amount of IgA-coated bacteria was significantly higher in IL-10^−/−^ mice compared with wild-type mice (80.5 ± 12.10 vs. 54.8% ± 2.12%, *p* = 0.001), however, in sodium butyrate-treated mice, the amount of IgA-coated bacteria was significantly decreased (44.7 ± 0.92 vs. 80.5% ± 12.10%, *p* < 0.001), and there was no significant difference between wild-type mice and the IL-10^−/−^ mice receiving sodium butyrate treatment (*p* = 0.087).

## 4. Discussion

Our study shows that sodium butyrate ameliorated histological colitis which was associated with a reduction in the amount of IgA-coated bacteria, modified the composition of the microbiome and changed the SCFAs concentration in the colon in IL-10^−/−^ mice.

Many studies have revealed effects of soluble dietary fiber on inflammation in IBD [15,16,28] but the mechanisms behind such effects remain largely unknown. SCFAs, fermented from soluble fiber by certain species of gut bacteria, have a crucial role as a fuel source for intestinal epithelial cells [17] and exert effects on both gut morphology and function [18]. Previous reports showed a decrease in sodium butyrate after feeding rats a diet containing 5% pectin [29,30]; however, other studies have shown an increase in sodium butyrate concentration after feeding apple pectin to animals included rats, pigs and mice [31,32,33]. In the current study, significantly higher concentrations of acetate, propionate and sodium butyrate were observed in the IL-10^−/−^ mice without treatment compared with the IL-10^−/−^ mice receiving sodium butyrate, which is consistent with a report that the concentration of SCFAs in mice with dextran sulfate sodium (DSS)-induced colitis treated with sodium butyrate were lower than those without treatment [30]. These inconsistencies suggest that SCFAs were lost or not totally absorbed in the damaged gut mucosa of IL-10^−/−^ mice and sodium butyrate increased metabolism or absorption of SCFA in the colon. 

Sodium butyrate-producing bacteria, including *Clostridium*, *Eubacterium* and *Fusobacterium*, mainly live in the caecum and colon. A Japanese study found that sodium butyrate-producing bacterial species, such as *Blautia faecis*, *Roseburia inulinivorans*, *Ruminococcus torques*, *Clostridium lavalense*, *Bacteroides uniformis* and *Faecalibacterium prausnitzii* were significantly reduced in CD patients compared with healthy individuals [34]. A reduction in *Roseburia hominis* and *F. prausnitzii*, both well-known sodium butyrate-producing bacteria of the *Firmicutes* phylum, was also observed in ulcerative colitis patients [22]. It was also found that SCFAs were reduced, but no direct correlation between SCFA and the identified bacteria was found. Depletion of sodium butyrate-producing bacteria in IBD microbiota is clearly evidenced by a reduction in sodium butyrate-producing metabolic pathways [19,20], as well as change in concentrations of fecal sodium butyrate [21,22]. We found that levels of sodium butyrate-producing *Eubacterium* bacteria in wild-type mice were significantly higher than other groups. However, levels of sodium butyrate-producing *Clostridium* bacteria in IL-10^−/−^ mice receiving sodium butyrate were higher than wild-type mice. This trend is consistent with the concentration of sodium butyrate. Therefore, these results suggest that altering the microbiota (resulting in more butyrate-producing bacteria) would be an alternative long-term treatment option to ongoing supplementation with butyrate. The paradoxical results might be explained by the limited numbers of mice per group or the dysbiosis of the gut microbiota. Therefore, further investigation will be needed to confirm this hypothesis.

Studies suggest that dysbiosis of the gut microbiota, as characterized by decreases in the proportions of *Firmicutes* and *F. prausnitzii* and increases in *Bacteroidetes* and *Enterobacteriaceae* [4,35,36] is a major characteristic of IBD. The effects of the composition of the intestinal microbiota on disease progress in mouse models of IBD have been examined in studies which found particular bacterial taxa within the intestinal microbiota, such as *Prevotellaceae* [37,38] and *Helicobacter* species [39], can drive chronic intestinal inflammation or exacerbate colitis. Furthermore, a study reported that bacteria isolated from patients with IBD and selected based on high IgA-coating induced potent IgA responses and dramatically exacerbated the development of DSS-induced colitis in gnotobiotic mice [9]. In the present study, we found that sodium butyrate reduced the amount of IgA-coated bacteria in IL-10^−/−^ mice. IgA is prominently secreted at mucosal surfaces and coats a fraction of the intestinal microbiota [40]. IgA can mediate protective immunity to enteric pathogens including viruses, bacteria, and toxins [41]. Moreover, one study found that sodium butyrate regulates the size and function of the colonic Treg pool and protects against colitis in a GPR43-dependent manner in mice [23]. Therefore, sodium butyrate might reduce the amount of IgA-coated bacteria through improving protective immunity to enteric pathogens.

In the current study, dysbiosis of the gut microbiota was regulated by oral administration of sodium butyrate in IL-10^−/−^ mice, specifically an increase in the proportion of *Firmicutes* and *Lactobacillus* and decrease in *Bacteroidetes* and *Prevotella.* Furthermore, it also modified the abundance of the gut microbiota. Previous studies found decreased biodiversity of microbiota in IBD patients and mice [5,42,43,44,45] and a clinical trial showed that ulcerative colitis patients receiving fecal microbiota transplantation had greater microbial diversity than those given the placebo, and more patients who had greater microbial diversity went into remission [46]. Therefore, we consider modifying dysbiosis and enriching biodiversity might be important mechanisms in sodium butyrate amelioration of colitis in IL-10^−/−^ mice. 

This study has several limitations. First, though the results are in accord with our hypothesis, there were no significant differences in some bacteria when analyzing the composition of microbiota, which might be due to the limited number of mice per group. Second, a similar clinical study performed in patients with IBD needs to be undertaken. Last, a rigorous causal relationship between colitis and the composition of microbiota in this study was not confirmed. 

## 5. Conclusions

In summary, our data provide evidence that oral treatment with sodium butyrate protects against colitis, possibly through reducing the amount of colitogenic IgA-coated bacteria and modifying gut microbiota in IL-10^−/−^ mice. An increased understanding of the protective mechanism of sodium butyrate might shed light on potential novel therapeutic options in patients with IBD.
